# Potential Mechanisms of Abiotic Stress Tolerance in Crop Plants Induced by Thiourea

**DOI:** 10.3389/fpls.2019.01336

**Published:** 2019-10-29

**Authors:** Muhammad Ahmed Waqas, Cengiz Kaya, Adeel Riaz, Muhammad Farooq, Iqra Nawaz, Andreas Wilkes, Yue Li

**Affiliations:** ^1^Institute of Environment and Sustainable Development in Agriculture, Chinese Academy of Agricultural Sciences, Beijing, China; ^2^Laboratory for Agricultural Environment, Ministry of Agriculture, Beijing, China; ^3^Department of Soil Science & Plant Nutrition, Faculty of Agriculture, Harran University, ¸Sanlıurfa, Turkey; ^4^Biotechnology Research Institute, Chinese Academy of Agricultural Sciences, Beijing, China; ^5^Department of Crop Sciences, College of Agricultural and Marine Sciences, Sultan Qaboos University, Al-Khoud, Oman; ^6^Department of Agronomy, University of Agriculture, Faisalabad, Pakistan; ^7^UWA Institute of Agriculture and School of Agriculture & Environment, The University of Western Australia, Perth, WA, Australia; ^8^Institute of Pomology, Chinese Academy of Agricultural Sciences, Xingcheng, China

**Keywords:** heat, cold, heavy metal, drought, salinity, antioxidants, osmolytes biosynthesis, climate change

## Abstract

Abiotic stresses, such as temperature extremes, drought, salinity, and heavy metals are major factors limiting crop productivity and sustainability worldwide. Abiotic stresses disturb plant growth and yield formation. Several chemical compounds, known as plant growth regulators (PGRs), modulate plant responses to biotic and abiotic stresses at the cellular, tissue, and organ levels. Thiourea (TU) is an important synthetic PGR containing nitrogen (36%) and sulfur (42%) that has gained wide attention for its role in plant stress tolerance. Tolerance against abiotic stresses is a complex phenomenon involving an array of mechanisms, and TU may modulate several of these. An understanding of TU-induced tolerance mechanisms may help improve crop yield under stress conditions. However, the potential mechanisms involved in TU-induced plant stress tolerance are still elusive. In this review, we discuss the essential role of TU-induced tolerance in improving performance of plants growing under abiotic stresses and potential mechanisms underlying TU-induced stress tolerance. We also highlight exploitation of new avenues critical in TU-induced stress tolerance.

## Introduction

Abiotic factors are the major yield-limiting factors for crop plants (Canter, 2018; Zörb et al., 2019). Temperature extremes, drought, flooding, salinity, and heavy metal stress, among others, affect the growth and yield formation of crop plants (Waqas et al., 2017; Vaughan et al., 2018; Zafar et al., 2018). About 90% of arable lands are prone to one or more of the above stresses (dos Reis et al., 2012), which cause up to 70% yield losses in major food crops (Mantri et al., 2012). Estimates based on the integration of climate change and crop yield models have predicted further loss in the productivity of major crops, including rice, wheat, and maize, which may have serious consequences for food security (Tigchelaar et al., 2018).

The extent of salinity in irrigated lands has increased by 37% during the last two decades (1990–2013) (Ghassemi et al., 1995; Qadir et al., 2014). Changes in the patterns of precipitation and global warming-induced increase in evapotranspiration have increased the frequency and severity of drought stress (Dai, 2011). A recent meta-analysis study indicated an increase of 2.0 to 4.9°C by 2100 in global average temperature (Raftery et al., 2017). Increasing contamination of arable lands with heavy metals is not only limiting crop productivity but is also causing serious risks to human health (Rehman et al., 2018).

Plant growth regulators (PGRs) modulate plant responses to biotic and abiotic stresses and regulate their growth and developmental cascades. Thiourea (TU), a synthetic compound containing nitrogen (as -NH_2_) and sulfur (as -SH), is an important PGR, which influences plant growth particularly under stress conditions (Garg et al., 2006). Since the discovery of the biological roles of TU (Jocelyn, 1972), it has only been employed to break seed dormancy and improve germination (Esashi et al., 1977). However, increasing research interest in TU has recently revealed several beneficial impacts on plant biology.

Exogenous application of TU (e.g. as seed priming, foliar spray, medium supplementation, soil application) stimulates defense mechanisms in plants under abiotic stress (Akladious, 2014; Srivastava et al., 2017; Seleiman and Kheir, 2018). TU application modulates key physiological events and mechanisms, including photosynthesis, nitrogen metabolism, proline metabolism, antioxidant defense systems, and plant water relations during different plant developmental stages (Kaya et al., 2015; Vineeth et al., 2016; Wakchaure et al., 2018; Kaya et al., 2019). TU application up-regulates the expression of genes involved in encoding reactive oxygen species (ROS)-activated ion channels, antioxidants, regulation of redox state, aquaporins, osmotic adjustment, metabolite biosynthesis, calcium signaling, and hormonal regulation, such as catalase and cytochrome P450 (Srivastava et al., 2009; Demidchik et al., 2010; Srivastava et al., 2011; Patade et al., 2012). The application of TU also modulates post-transcriptional regulation to enhance the expression of defense-related genes by the synchronization of microRNAs and hormones (Srivastava et al., 2017).

Plant responses to TU vary by plant species and depend on the concentration applied. For instance, in potato (*Solanum tuberosum*) a lower concentration of TU (250, 500 mM) increased carotenoid, ascorbic acid, polyphenol, and nitrate contents, while a higher concentration (1,000 mM) significantly decreased nitrate content (Mani et al., 2012). However, in lentil (*Lens culinaris*) grown under rain-fed conditions, a higher concentration of TU (13 mM) significantly enhanced seed yield, quality and economic returns of the crop when compared with a low concentration (6.5 mM) of TU (Premaradhya et al., 2018). High doses (such as 60 mM) can cause more severe injury to wheat (*Triticum aestivum*) plants compared with low doses (20 mM and 40 mM) (Suryavanshi et al., 2016). Besides the dose applied, plant species, growth stage, climatic conditions, treated plant organ, duration of treatment, and application method also affect the ability of TU to alleviate abiotic stress damage (Kaya et al., 2013; Waqas et al., 2017; Seleiman and Kheir, 2018).

These shreds of evidence indicate that abiotic stress tolerance is an intricate phenomenon involving many metabolic, cellular, and molecular aspects (Sasaki, 2006; Hussain et al., 2018; Ronen et al., 2019). Due to the limited success achieved with conventional breeding in developing and least-developed regions, abiotic stresses are increasingly threatening sustainable agricultural development. Therefore, to improve plant performance and economic returns to farmers amidst the ongoing challenge of a rapidly changing climate, it is important to investigate new avenues for TU-induced tolerance in crop plants. However, the published reviews only focused either on crop productivity gains, or biological changes with TU application (Pasala et al., 2016; Wahid et al., 2017). Thus, this review discusses the biological role of TU in inducing tolerance against abiotic stresses (e.g. temperature extremes, drought, salinity, and heavy metal stress) in crop plants. Potential mechanisms of TU-induced tolerance, and potential new avenues for exploiting TU-induced stress tolerance are also described.

### Temperature Stresses

Cold, chilling, and heat stress during critical stages of plant development have strong impacts on developmental cascades of crop plants (Zhou et al., 2018). Temperature stress reduces the plant's optimal biochemical and physiological functioning by modulating molecular mechanisms (Djanaguiraman et al., 2018; Muhlemann et al., 2018; Takahashi and Shinozaki, 2019). For instance, jatropha (*Jatropha curcas*) is an important bioenergy crop, but it's ability to produce biofuel is often restricted under cold stress (Wang et al., 2013). However, TU (1.3 mM) seed priming enhanced cold tolerance by decreasing leaf senescence and maintaining the relative water content in jatropha plants grown at 4°C (Yadav et al., 2012). Low-temperature stress not only decreases grain yield but also affects crop grain quality (Dreccer et al., 2018). However, TU (2.6 mM) foliar spray applied at the six-leaf and tasseling stages were effective in improving grain yield and the quality of maize (*Zea mays*). These improvements were attributed to better growth, and higher chlorophyll content, rate of photosynthesis, and dry matter accumulation under chilling stress (Waqas et al., 2017). In bell pepper (*Capsicum annuum*), TU primed seed had an improved germination rate under cold stress (15°C) and subsequent seedlings from primed seed exhibited significantly better survival rates under cold stress (4°C) (Yadav et al., 2011). Stress tolerance mechanism in plants is regulated *via* transcriptional activation or repression. Evidence suggests that TU-induced cold tolerance is linked with the increased expression of genes involved in transcriptional regulation (*CaWRKY_30_*), osmotic adjustment (PROX_1_ and Osmotin), antioxidant defense (Cu/Zn SOD), and metabolite biosynthesis (CAH) (Patade et al., 2012).

TU supplementation has also been reported to mitigate the adverse impacts of heat stress in crop plants (Asthir et al., 2013; Khanna et al., 2017). Seed pre-treatment (6.6 mM) and foliar spray of TU (6.6 mM) at the anthesis stage were effective in improving tolerance against terminal heat by lowering oxidative damage (cell membrane injury, thiobarbituric acid reactive substances, and H_2_O_2_ contents) and increasing the antioxidant potential in wheat under heat stress (29.4°C) at the grain filling stage (Asthir et al., 2013). TU seed treatment (10 mM) led to significant improvement in achene yield and oil content in sunflower (*Helianthus annuus*) under mild and extreme heat stress (35°C and 45°C) by inducing antioxidant defense (SOD, CAT, and APX), and by maintaining leaf water and nutrient status (N, P, K), nitrate reductase and phenylalanine ammonia lyase activity, and the rate of photosynthesis (Akladious, 2014). DNA profiling using random amplified polymorphic DNA (RAPD) after TU application indicated significant variations in sunflower plants. More than 87% of DNA bands were polymorphic and identified positive markers for thermotolerance. The identified positive markers could be used to identify genes associated with heat tolerance in marker-assisted breeding.

Prolific root systems and optimal grain filling are crucial to improve stress tolerance and enhanced final yield (Palta and Turner, 2018). TU (10 mM) increased root growth and grain filling rate to impart heat-tolerance in bread wheat (Freeha et al., 2011). In wheat plants growing under 7–10°C higher than ambient temperature, TU improved flag leaf gas exchange and water use efficiency by increasing CO_2_ uptake of Rubisco (Freeha et al., 2008). Application of TU increased K^+^ uptake and reduced ABA biosynthesis in chickpea under heat stress (Aldasoro et al., 1981). TU application also modulates carbon metabolism to improve heat tolerance (Asthir et al., 2015). TU foliar spray (20 mM) at anthesis significantly improved the grain yield (+14.9%) of a field-grown wheat crop exposed to terminal heat stress (2-3°C higher than ambient) in the northwest of India (Suryavanshi et al., 2016). In maize, TU spray (6 mM) upregulated the activities of catalase and peroxidase in roots and shoots, H_2_O_2_ scavenging efficiency, differential biomass partitioning, and an active ascorbate-glutathione cycle to ameliorate the negative impacts of extreme temperature stress (40°C) (Khanna et al., 2017). Beyond this current evidence, there is still a need to evaluate the detailed molecular mechanisms of TU-induced high-temperature tolerance.

### Drought Stress

Rapidly changing climate dynamics makes drought a serious threat to the sustainability of food production systems throughout the world (Kogan et al., 2019). For example, in southern Europe, general decreases in crop yield (e.g. legumes –30 to +5%, sunflower –12 to +3%, and tuber crops –14 to +7%) and increases in water demand (e.g. maize +2 to +4%, potato +6 to +10%) are expected for spring-sown crops by 2050 (Skuras and Psaltopoulos, 2012). Drought stress strongly influences plant growth and yield formation (Sperry et al., 2002; Tardieu et al., 2017; Bartlett et al., 2019).

Plants adapt in various ways in response to drought stress, such as alterations in growth pattern, plant morphology, and defense mechanisms (Zandalinas et al., 2018). Exogenous TU application has been shown to improve plant defense systems to a significant extent, which helps improve phloem translocation of photosynthates in crop plants and thereby induces drought and salinity tolerance in cereals, pulses, and oilseeds (Srivastava et al., 2009; Bhunia et al., 2015; Singh and Singh, 2017). Leaf relative water content (LRWC) is an index of tissue water status. Drought causes a decrease in LRWC (Dias et al., 2018). Exogenous spray of 10 mM TU at critical growth stages of wheat (i.e., crown root initiation, flag leaf, and seed milking) significantly increased water productivity by maintaining higher LWRC and modulating stomatal opening, which enabled plants to make better use of water under medium (irrigation water/cumulative pan evaporation ratio (IW: CPE) 0.40–0.69) and severe (IW: CPE 0.10–0.39) stress conditions (Pasala, 2017).

TU application reduces water consumption by plants under water deficit conditions. Wakchaure et al. (2016) studied the effect of TU application to wheat (*Triticum aeastivum*), and concluded that the maximum water productivity obtained without TU could be obtained with 19–56% less water when TU was applied. Integrated use of TU *via* seed treatment (500 ppm) and foliar application (1,000 ppm) at the pre-flowering stage of mung bean (*Vigna radiata*) increased seed yield (24%) by improving photosynthetic efficiency and plant metabolic functioning under rainfed conditions (Mathur et al., 2006). TU also enhanced the water use efficiency, growth, and economic yield of cluster beans (*Cyamopsis tetragonoloba*) by improving nitrogen metabolic aspects, especially nitrate reductase activity, and by more efficient photosynthesis under water stress (Garg et al., 2006). TU-treated plants showed better performance regarding plant growth despite experiencing slightly more water deficit due to stabilization of lipoprotein structure and less malondialdehyde production compared to control plants (Mohammadi and Karr, 2001). In wheat, plants irrigated at 40% field capacity drastically showed detrimental effects on physiological and biochemical processes, including translocation, ion uptake, respiration, photosynthesis, carbohydrates, nutrient metabolism, and hormones (Abdelkader et al., 2012). However, seed pre-treated with salicylic acid (∼1 mM) and two foliar applications of TU (5 mM) before anthesis significantly enhanced grain yield and quality (Abdelkader et al., 2012). In addition, studies have reported that although there can be a significant increase in MDA and H_2_O_2_ in response to drought stress, which causes oxidative damage due to excessive production of ROS, TU application scavenges ROS by improving antioxidant defense (Hassanein et al., 2015). Another system is lipid peroxidation (LPO), which is likely due to oxidative damage induced by drought stress. Chickpea plants pre-treated at the vegetative stage with TU (1,000 mg L^−1^) prior to drought stress had significantly lower LPO and improved quantum efficiency of photosystem II (Vineeth et al., 2016).

TU application regulates the expression of a group of genes known as 'thiourea responsive genes' which are related to oxidative stress, calcium signaling, and hormonal regulation. These include catalase and cytochrome P450, calcium-transporting ATPase, calcium exchanger, and calmodulin-like proteins (Srivastava et al., 2009). Water stress lowers the expression of photo-respiratory genes and Rubisco large subunit (*RbcL*) related genes as part of the response by the plant defensive system. TU treatment upregulates the expression of genes like *RbcL*, and glycolate oxidase and glycine decarboxylase H subunits, to guard the photosynthesis factory of plants under drought stress (Vineeth et al., 2016). Drought stress severely hampers water productivity in crops. Recently, Wakchaure et al. (2018) described that TU application also improves water productivity, commercial quality, and bulb yield of onion (*Allium cepa*), leading to a 48.4% reduction in water consumption under scarce water conditions. Evidence suggests that TU application upgrades several metabolic functions to induce drought tolerance (**Table 1**).

**Table 1:** Influence of TU application on tolerance against abiotic stresses in crop plants.

Plant	Concentration	Influence on plant	Response	Reference
**Heat stress**				
Wheat	6.5 mM	Reduced cell membrane injury	+	(Asthir et al., 2013)
Sunflower	10 mM	Modulation in plant protein pattern	+	(Akladious, 2014)
Wheat	10 mM	Enhanced Rubisco activity and water use efficiency	+	(Freeha et al., 2011)
Wheat	7 mM	Modifications in carbon metabolism	+	(Asthir et al., 2015)
Maize	6 mM	More active ascorbate–glutathione cycle	+	(Khanna et al., 2017)
**Cold stress**				
Jatropha	1.3 mM	Decreased leaf senescence and membrane damage	+	(Yadav et al., 2011)
Maize	2.6 mM	Improved photosynthetic activity	+	
Capsicum	1.3 mM	Enhanced the expression of defense-related genes		(Patade et al., 2012)
**Drought stress**				
Wheat	10 mM	Modulated stomatal opening and closing	+	(Wakchaure et al., 2016)
Mung bean	13.6 mM	Increased plant metabolic functioning	+	(Mathur et al., 2006)
Onion	6.5 mM	Improved water productivity	+	(Wakchaure et al., 2018)
Chickpea	13.6 mM	Increased expression of RbcL and glycolate oxidase to maintain photosynthesis	+	(Vineeth et al., 2016)
Beans	13.6 mM	Improved nitrogen metabolic activity	+	(Garg et al., 2006)
**Lead stress with acid rain**				
Fenugreek	3 mM	Up-expression of SOD and CAT genes.	+	(Xalxo and Keshavkant, 2019)
**Cadmium stress**				
Maize	0.25 mM	Reduced Cd-availability to the root and its transport to shoot	+	(Perveen et al., 2015)
Barley	10 mM	Reduced oxidative damage	+	(Ikram et al., 2014)
**Arsenic stress**				
Rice	75 μM	Orchestrated redox homeostasis	+	(Srivastava et al., 2014)
Lentil	13 mM	Seized As in roots to prevent movement to other organs	+	(Talukdar, 2017)
**Boron Toxicity**				
Wheat	5.26 mM	Stimulated nitric oxide production	+	(Kaya et al., 2019)
**Salinity stress**				
Indian mustard	6·5 mM	Regulated different signaling and effector mechanisms	+	(Srivastava et al., 2010)
Maize	6.5 mM	Improved K+/Na+ ratio, uptake of K+, Ca2+, and N and water relations	+	(Kaya et al., 2015)
Mung bean	20 mM	Enhanced glycine betaine contents	+	(Perveen et al., 2016)
Wheat	240 g ha−1	Improved nutrient uptake and photosynthetic machinery	+	(Seleiman and Kheir, 2018)
Maize	6.5 mM	Decreased leaf Na+ and membrane damage	+	(Kaya et al., 2016)

### Heavy Metal/Metalloids

Plants largely depend on soil solution to acquire nutrients for their growth and developmental cycle. The recent increase in contamination of arable lands with heavy metals is one of the most important causes of loss in crop productivity (Proshad et al., 2018). Extensive exposure to heavy metal contamination threatens the sustainability of environmental and agricultural systems. Crops are routinely subjected to metal toxicity due to improper irrigation methods and the addition of excessive quantities of chemical fertilizers, and other synthetic nutrients (Pasala, 2017). Industrial and sewage waste disposal, urban runoff, burning of solid and liquid fuels, domestic garbage disposal in rivers and canals, and many other pathways cause heavy metal contamination. Some (potentially toxic) heavy metals, such as Cu, Zn, Ni, Co, Se, and Fe, are also essential elements required for the optimal performance of plants and become toxic when accumulated in excess in soil solution (Khan et al., 2018; Narendrula-Kotha et al., 2019). On the other hand, non-essential elements, such as arsenate (As), cesium (Cs), lead (Pb), and cadmium (Cd), can hamper crop productivity when accumulated in the soil even in trace amounts (Khalid et al., 2018). Soil contamination with heavy metals causes accumulation of these toxic metals in plant parts, resulting in decreased crop productivity and increased risk to animal and human health (Couto et al., 2018).

Cd stress reduces plant growth, as evident from plant stunting, reduced leaf area, and decreased shoot and root dry matter yield (Shah et al., 2019). However, root applied TU (0.25 mM) induced Cd tolerance in maize plants grown in pots supplemented with nutrient solution and sand by improving leaf expansion, photosynthetic rate, stomatal conductance, Chl-b, and carotenoids contents, and reducing Cd-availability to the root and its transport to shoots (Perveen et al., 2015). Exogenous application of TU ameliorated the effects of Cd stress in barley (*Hordeum vulgare*) by improving relative fresh and dry weights as well as reducing oxidative stress (Ikram et al., 2014).

**Figure 1:**
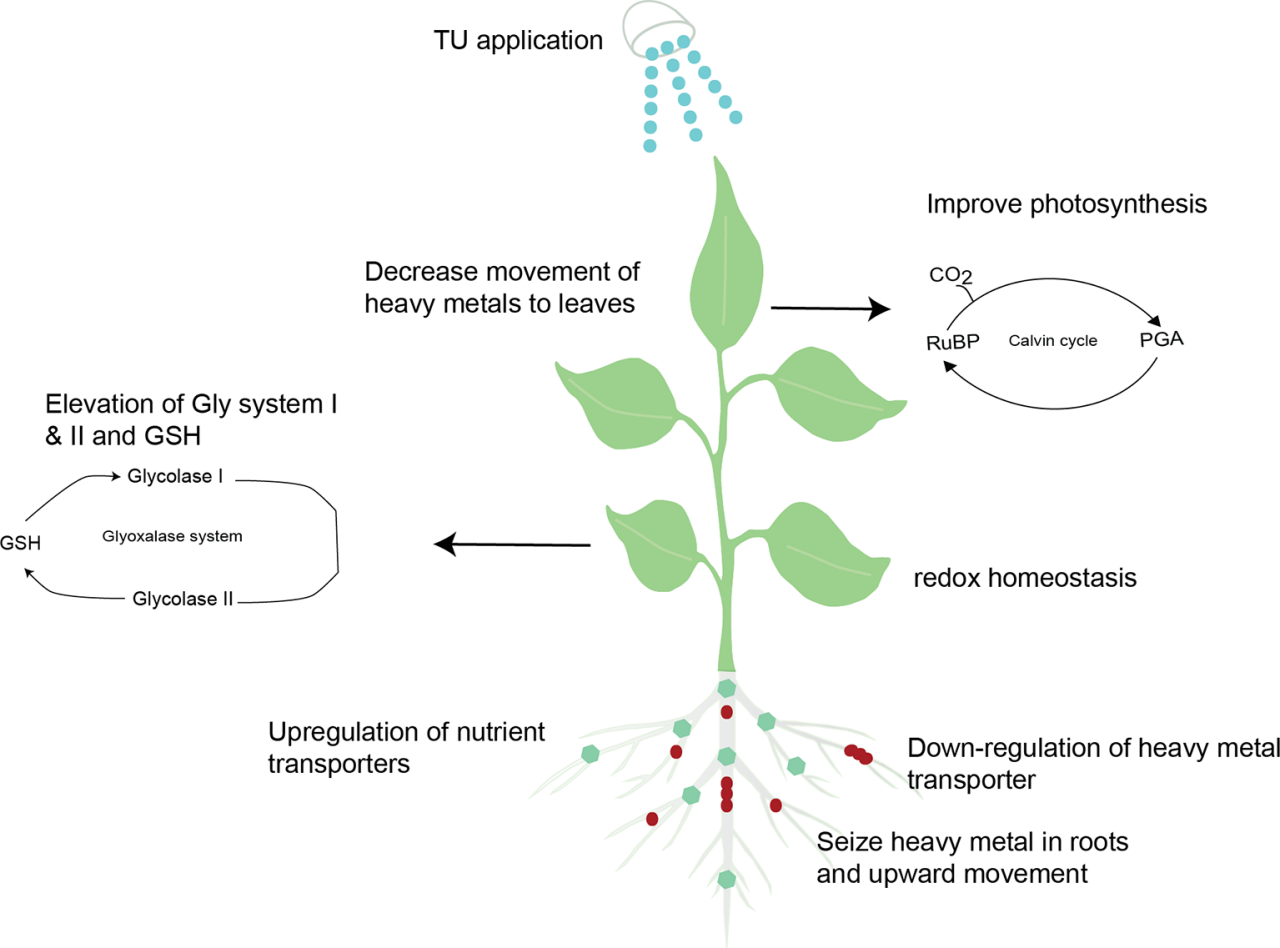
Schematic illustration indicating TU-mediated heavy metal tolerance in plants.

Arsenic (As) is a carcinogenic element present worldwide that harmful to every form of life (Ghosh and Mukhopadhyay, 2019). TU orchestrates redox homeostasis to impart tolerance against heavy metal stress. TU-mediated redox homeostasis triggered the downregulation of Lsi2 (transporters for As translocation) which reduced As levels by 56%. Concurrently, up-regulation of Sultr1;1 and 1;2 (sulfate transporters) enhanced sulfur metabolism to ameliorate the adverse effects of As stress (Srivastava et al., 2014). Through these mechanisms, TU restricts heavy metal translocation to shoots and sequesters them in roots (**Figure 1**). Another possible mechanism of reduced loading of metal ions in xylem from roots to shoots with TU supplementation is chelation of metal ions with thiols (TU contains thiol- group) in the cytosol. Presence of thiols facilitates efficient utilization of SH-containing compounds as biomarkers of metal tolerance (Borisova et al., 2016; Meng et al., 2019). Thiols also permit vacuolar compartmentation (Guo et al., 2012), which might be a vital mechanism through which TU reduces metal-toxicity, but this has not yet been experimentally proven.

Further, TU modulates glyoxalase system I and II and ascorbate (AsA)-glutathione (GSH) redox, and antioxidant defense enzymes in both leaves and roots of As-exposed plants. Elevation of the Gly system prevents toxic methylglyoxal overaccumulation, whereas more active AsA-GSH cycle enzymes and GSH-s-transferase, and catalase effectively scavenge H_2_O_2_ to prevent ROS-mediated oxidative damage (**Figure 1**; Talukdar, 2016). In sum, it appears that the judicious application of TU can improve the performance of plants exposed to heavy metals and can help to decrease the load of metal ions in the edible parts of food crops.

### Salinity Stress

Salinity is increasingly becoming a major limiting factor to food security, as about 45 million hectares of irrigated land are under saline stress worldwide, and more than 50% of arable land could be salt-affected by the year 2050 (Munns and Tester, 2008). Most agricultural lands affected by salinity are located in semi-arid or arid regions. Therefore, the damage is intensified by the synchronized action of xerothermic aspects, such as aridity and high temperature (Huang et al., 2019). Salt stress stimulates ROS generation, that damages biomolecules (e.g., lipids, proteins, and nucleic acids) and alters redox homeostasis (Kundu et al., 2018). Plants employ different means to sense, respond, and adapt to changing the saline environment based upon modifications in morpho-physiological traits and ionic, biochemical, and molecular metabolisms which may further be improved by TU induction. Recent reports have evidenced the role of TU in improving the salt tolerance and underlying mechanisms in many plants, including Indian mustard (*Brassica juncea*) (Srivastava et al., 2009), *Halopyrum mucronatum* (Khan and Ungar, 2001), wheat (Seleiman and Kheir, 2018), *Aeluropus lagopodies* (Gulzar and Khan, 2002), maize (Kaya et al., 2016), and mung bean (Perveen et al., 2016).

Evidence suggests that TU treatment (6.5 mM) improved salt tolerance in *Brassica juncea* by enhancing the translocation of sucrose from source to sink (Srivastava et al., 2008). Recently, it has been discovered that mitochondria play a critical role in plant protection again salinity stress (Paiva et al., 2019). Application of TU to salt-stressed plants reverses the negative effects induced by salinity stress and thus maintains the effectiveness of mitochondria. This is an important mechanism by which TU maintains mitochondrial homeostasis and ATPases (FoF1-ATP synthase) plays an important role in TU-induced salt tolerance in *Brassica juncea* (Srivastava et al., 2009). TU application can also alleviate the adverse effects of salt stress by inducing changes in transcription through the modulation of microRNA and hormone production (Srivastava et al., 2017). A prolific root system is important to improve stress tolerance and final yield (Isayenkov and Maathuis, 2019). Exogenous TU application promotes root growth at seedling and pre-anthesis stages in wheat cultivars (Freeha et al., 2011). Positive impacts on root growth are likely due to an increase in leaf expansion, photosynthesis, sucrose movement from leaves to roots, and nutrient availability (as discussed in the previous sections). Moreover, TU improves the mechanisms underlying salt-tolerance by enhancing shoot-growth, chlorophyll content, and water relations, reduced EL and H_2_O_2_ content, increased K^+^/Na^+^ ratio, and increased uptake of K^+^, Ca^2+^, and N (Kaya et al., 2015). TU application (20 mM) significantly reduced the negative impacts of salt stress in mung bean by improving chlorophyll b, total soluble sugars, total soluble proteins, POD activity, and total phenolic contents, while decreasing membrane permeability, total free amino acids, and glycine betaine contents (Perveen et al., 2016). A recent study exhibited the very interesting phenomenon in response to application of soil bagasse ash (10 ton ha−1) and foliar TU (240 g ha−1) in saline soils of north and south Egypt. This combination proved successful in ameliorating negative impacts of salt stress and enhanced grain yield and quality (e.g. carbohydrate, fibre, protein, and ash contents of wheat) by improving nutrient uptake of N, P and K, EC, compaction, hydraulic conductivity and organic matter, as well as photosynthetic machinery (Seleiman and Kheir, 2018). Synchronous increase in uptake of N, P, K^+^, Ca^2+^ and decrease in Na^+^ uptake suggests that TU application induces salt avoidance tactics in plants under salinity stress.

### Potential Mechanisms Involved in TU-Induced Stress Tolerance

**Figure 2:**
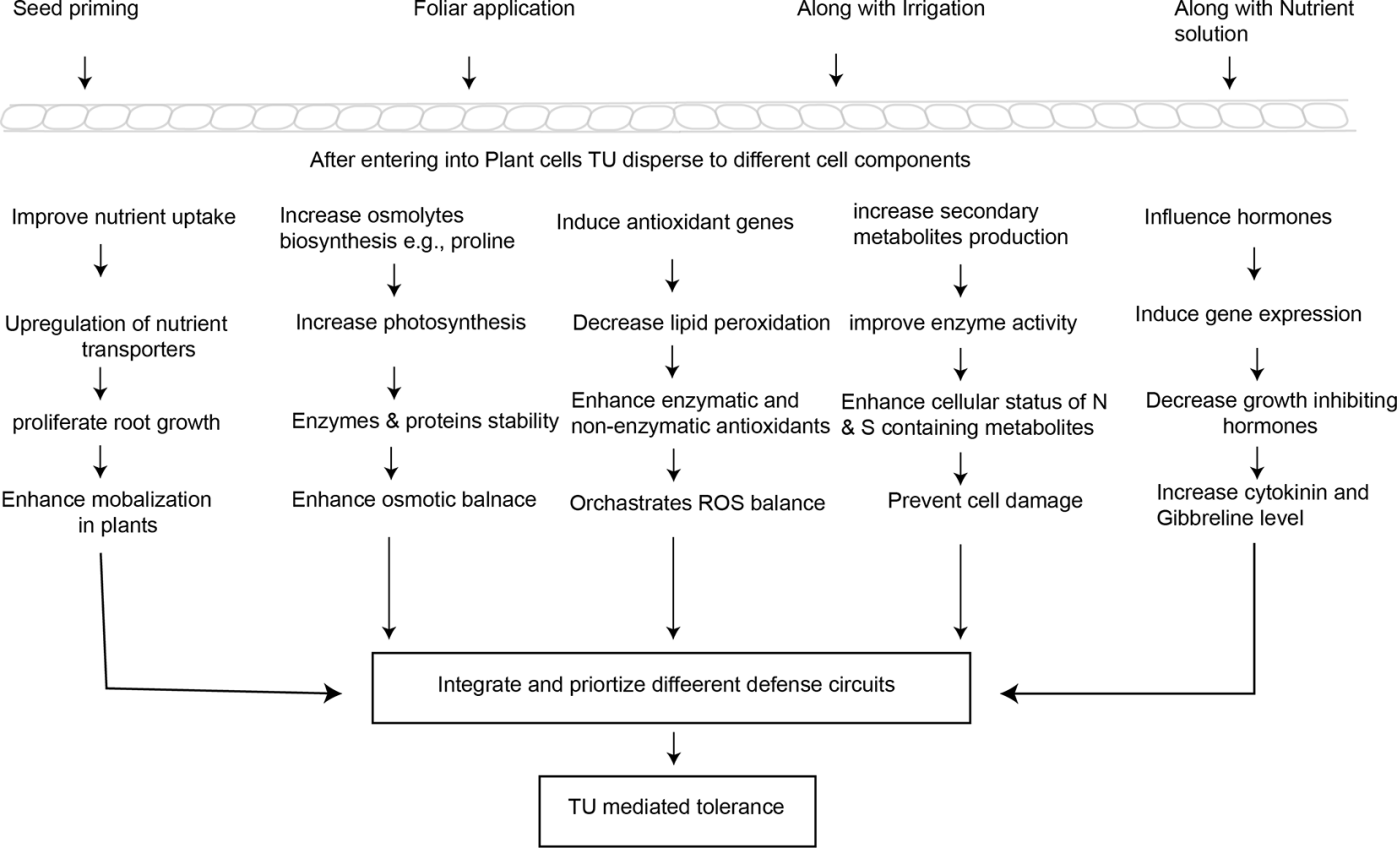
Simplified pattern indicating the potential mechanisms involved in TU-mediated abiotic stress tolerance and crop yield stability.

To explain the potential mechanisms underlying TU-induced tolerance in stressed plants, this section critically evaluates the recent literature on TU interactions with mineral nutrients and soil amendments, osmolytes, secondary metabolites, and with other plant hormones, and the critical role of TU in ROS-signaling and antioxidant defense (**Figure 2**).

### TU Improves Mineral Nutrition of Plants Under Stress Conditions

An adequate supply of mineral elements in the growth medium is required for plants to survive under environmental stresses. Plant nutrients status is related to their capacity to ameliorate the negative impacts caused by stress conditions (Hasanuzzaman et al., 2018; Wilkinson et al., 2019). As explained in above, TU is a rich source of N and S which could stimulate photosynthesis and thus proliferate root growth. Through this pathway, TU modulates nutrient uptake and assimilation, and plant metabolic processes to improve tolerance in stressed plants (Choudhary et al., 2017). TU (500 ppm) application twice at 45^th^ and 80^th^ days after sowing altered plant metabolism and enhanced nutrient concentrations (nitrogen, phosphorous, potassium, and sulphur) in both foliage and seed to improve the yield and quality of coriander (*Coriandrum sativum*) (Meena et al., 2015). In another study, TU (500 ppm) seed treatment and foliar application improved nitrogen (15%) and phosphorus (21%) uptake in cluster bean. Improved nutrient uptake enhanced the yield and quality (higher protein and gum content) of cluster bean under drought conditions (Meena et al., 2016). Beside root proliferation, evidence suggests that TU imparts changes in root physiology and biochemistry leading to better uptake of nutrients (N, P, K), resulting in an increase in yield and net-economic return (56%) under drought conditions in urdbean (*Vigna mungo*) (Choudhary et al., 2017). TU interacts positively with N and phosphorous to enhance the yield of water-stressed cluster bean by increasing net photosynthetic rates and concentrations of chlorophyll, starch, soluble protein, total free amino acids, and nitrate reductase activity compared to control plants at both vegetative and flowering stages (Burman et al., 2007). TU-mediated heat stress tolerance in sunflower was attributed to changes in leaf water, and nutrient status (N, P, K), nitrate reductase, and phenylalanine ammonia lyase (PAL) activities (Akladious, 2014), which are crucial enzymatic source of defense-related compounds in plants (Chamizo-Ampudia et al., 2017; Jun et al., 2018). Another possible mechanism of improved mineral nutrition is improvement in the water status (WS) of plants in response to TU (Waqas et al., 2017). Irrespective of nutrition source, a decline in WS elicits a decline in crop nutrient status (Kunrath et al., 2018). Overall, TU-mediated positive changes in root metabolism related to increased mineral nutrition are linked with more efficient photosynthesis machinery under environmental stress.

Similarly, foliar application of TU improved the grain yield of salt-stressed wheat and its protein concentration more than soil-applied bagasse ash (Seleiman and Kheir, 2018), mainly due to greater N uptake (Garg et al., 2006). Excess salinity causes excessive absorption of individual ions leading to toxicity and retards absorption of other essential plant nutrients leading to nutrient deficiencies (Sa et al., 2019). However, TU improves the mechanisms underlying salt-tolerance by modulating the K+/Na+ ratio and increasing uptake of K^+^, Ca^2+^, and N in maize plants (Kaya et al., 2016). TU also enhances the mobilization of essential nutrients in plants such as K^+^ and accumulates malate due to carboxylation of phosphoenol pyruvic acid with CO_2_ fixation in developing embryo (Hernández‐Nistal et al., 1983). TU had a positive impact in reducing the amounts of toxic ions such as Cd and increasing essential micro and macronutrient (e.g. Ca, Mg, Mn, N, S, etc.) contents, and simultaneously improves antioxidant defense and cell membrane stability to improve tolerance to Cd and saline stress (Ikram et al., 2014; Perveen et al., 2016). Inactivation/chelation and vacuolar compartmentation with TU application are plausible mechanisms for synchronous decrease in uptake or sequestration of toxic ions in roots and increase in uptake/mobilization of essential nutrients, but these mechanisms still need to be proven experimentally.

### Osmolyte Biosynthesis

To mitigate the negative effects of environmental stresses, plants increase the production of numerous compatible osmolytes, such as proline (Pro), glycine betaine (GB), amines, and soluble sugars. Such compounds assist in imparting tolerance in stressed plants by creating osmotic balance, membrane integrity, enzyme and protein stability, and ROS detoxification (Blum, 2017). Moreover, these compounds do not disturb normal plant functioning, but act as abiotic stress sensors and regulate cell volume in stressed plants (Singh et al., 2018; Takahashi and Shinozaki, 2019).

Extensive published data indicate the beneficial role of Pro accumulation in plants exposed to environmental stresses. Pro operates as a fine osmolyte, metal chelator, stress sensor, and antioxidant defender to impart tolerance in stressed plants (Ashraf et al., 2018; Singh et al., 2018). Numerous greenhouse and field experiments have shown that TU application, irrespective of the mode of application (e.g. seed treatment, foliar spray), enhances Pro metabolism in drought-stressed plants (Babar, 2013). TU application enhanced Pro accumulation (131%) in hybrid maize DK5783 and contributed to regulating leaf electrolyte, water potential, and osmolality under salt stress (Kaya et al., 2015). In contrast, another report showed that although TU improved salt tolerance and enhanced yield compared to a control treatment, it did not alter Pro accumulation significantly in salt-stressed mung bean (Perveen et al., 2016). These contrasting findings may be due to differences in experimental conditions, as the latter study was conducted in pots. Increase in Pro metabolism as a result of TU application was considered critical to alleviating heat stress effects in wheat by modulating chlorophyll content and photosynthetic rate (Suryavanshi and Buttar, 2018). Countries with a short winter duration do not offer conditions to meet the chilling requirements of buds of temperate perennial fruit trees (such as apple, kiwifruit) to release them from dormancy (Heide and Prestrud, 2005). Therefore, delay in bud break until late winter exposes them to high temperatures leading to low yield and quality. TU-mediated early bud break set high percentages of fruit by regulating the Pro content in apple buds (El-Yazal and Rady, 2013). More active oxidative pentose-phosphate pathway (PPP) in response to exogenous TU application may trigger Pro metabolism. Because PPP is a major source of NADPH in non-photosynthesis tissues (Corpas and Barroso, 2018), the resultant NADPH needs to be consumed to maintain normal functioning of PPP. Therefore, its utilization is carried out by reducing Δ^1^-pyrroline-5-carboxylate into proline (Miller et al., 2009). In general, the evidence indicates that TU-induced Pro biosynthesis is a vital mechanism in imparting tolerance in plants growing under unfavorable conditions. However, the signaling events, enzymes, genes, and transcriptional controls involved in TU-mediated Pro biosynthesis need to be further explored.

Another major osmolyte is GB which activates plants to protect themselves, especially in moisture stress conditions. GB mainly functions in the chloroplast to guard the thylakoid membrane against stress-induced damage and regulates photosynthetic apparatus (Kurepin et al., 2017). Only one study has reported the effects of TU application on GB accumulation. According to Perveen et al. (2016), salt-induced stress in mung bean significantly enhanced GB biosynthesis, which is dependent on plant species and even varied among different varieties. Enhanced synthesis of soluble sugars, mannitol, and polysaccharides has also been found to impart tolerance in stressed plants (Ceusters et al., 2016; Liu et al., 2019). TU treatment (20 mM) significantly reduced the negative impacts of salt stress by increasing total soluble sugars and proteins in mung bean plants (Perveen et al., 2016). TU also interacts positively with nutrients (nitrogen and phosphorous) to alleviate moisture stress by increasing starch, soluble protein, total free amino acids, and nitrate reductase activity in clusterbean plants in a dose-dependent manner (Burman et al., 2007). More active carbon and nitrogen metabolisms (Burman, 2004;Garg et al., 2006) in response to exogenous TU supply triggers the production and accumulation of these favorable compounds. Although TU-induced osmolyte biosynthesis is a critical mechanism in improving stressed plant performance, much more effort should be devoted to exploring the interactions between TU and osmolytes in order to enhance understanding of plant adaptive mechanisms.

### Role of TU in ROS-Detoxification and Antioxidant Defense Under Stress Conditions

Production and utilization of various ROS, such as O_2_^−^, OH^•^, H_2_O_2_, are normal functions in plant metabolism. Under environmental stresses, distortion of equilibrium brings many harmful consequences for plant metabolism and overall productivity (Czarnocka and Karpiński, 2018). ROS species can interplay with cell components, giving rise to peroxidative reactions. This scenario causes significant damage to lipids, proteins, nucleic acids, and in worst-case scenarios, even cell death and stunted plant growth (Dangol et al., 2019). Therefore, the balance between ROS production and utilization is critical for optimal plant growth.

TU-mediated stress tolerance could be related to its strong capacity for ROS detoxification, which was first observed in HL 60 cell lines (Kelner et al., 1990). Later on, TU has been proven to be a strong ROS-scavenger and antioxidant defender under numerous environmental stresses (Vineeth et al., 2016; Srivastava et al., 2017; Talukdar, 2017). For instance, drought and salinity cause oxidative damage by excessive production of ROS. Exogenous TU scavenged the ROS in drought-stressed wheat by enhancing antioxidant defense (Hassanein et al., 2015). Additionally, LPO is a key mechanism which can be taken as a signal of oxidative damage imposed by environmental stresses. TU treatment (13.6 mM) significantly lowered LPO and improved the quantum efficiency of photosystem II in chickpea plants subjected to water stress (Vineeth et al., 2016). TU also induced the expression of TU responsive genes which are related to tolerance against oxidative stress in *Brassica juncea* under salinity stress (Srivastava et al., 2009). In another study, Srivastava et al. (2011) reported that TU supplementation brought down levels of ROS to near control values in salinity-stressed plants. These positive effects were attributed to an increase in 1,1-diphenyl-2-picrylhydrazyl radical scavenging activity and the activity of ascorbate oxidase, an important component of stress signaling (Srivastava et al., 2011). AsA and GSH are active redox compounds that assist in keeping a homeostatic balance of the cellular redox state and are critical in plant defense-mechanisms against stresses (Hoque et al., 2018). TU-stimulated glyoxalase system I and II and AsA-GSH redox, AsA-GSH cycle enzymes, GSH-s-transferase, and catalase triggered ROS-detoxification to effectively scavenge H_2_O_2_ in lentil plants when exposed to arsenic stress (Talukdar, 2017). Moreover, TU application (6 mM) upregulated the activities of catalase (CAT) and peroxidase (POX), improved H_2_O_2_ scavenging efficiency of the system, and an active AsA-GSH cycle to ameliorate the negative impacts of heat stress (40°C) in spring maize (Khanna et al., 2017).

**Figure 3:**
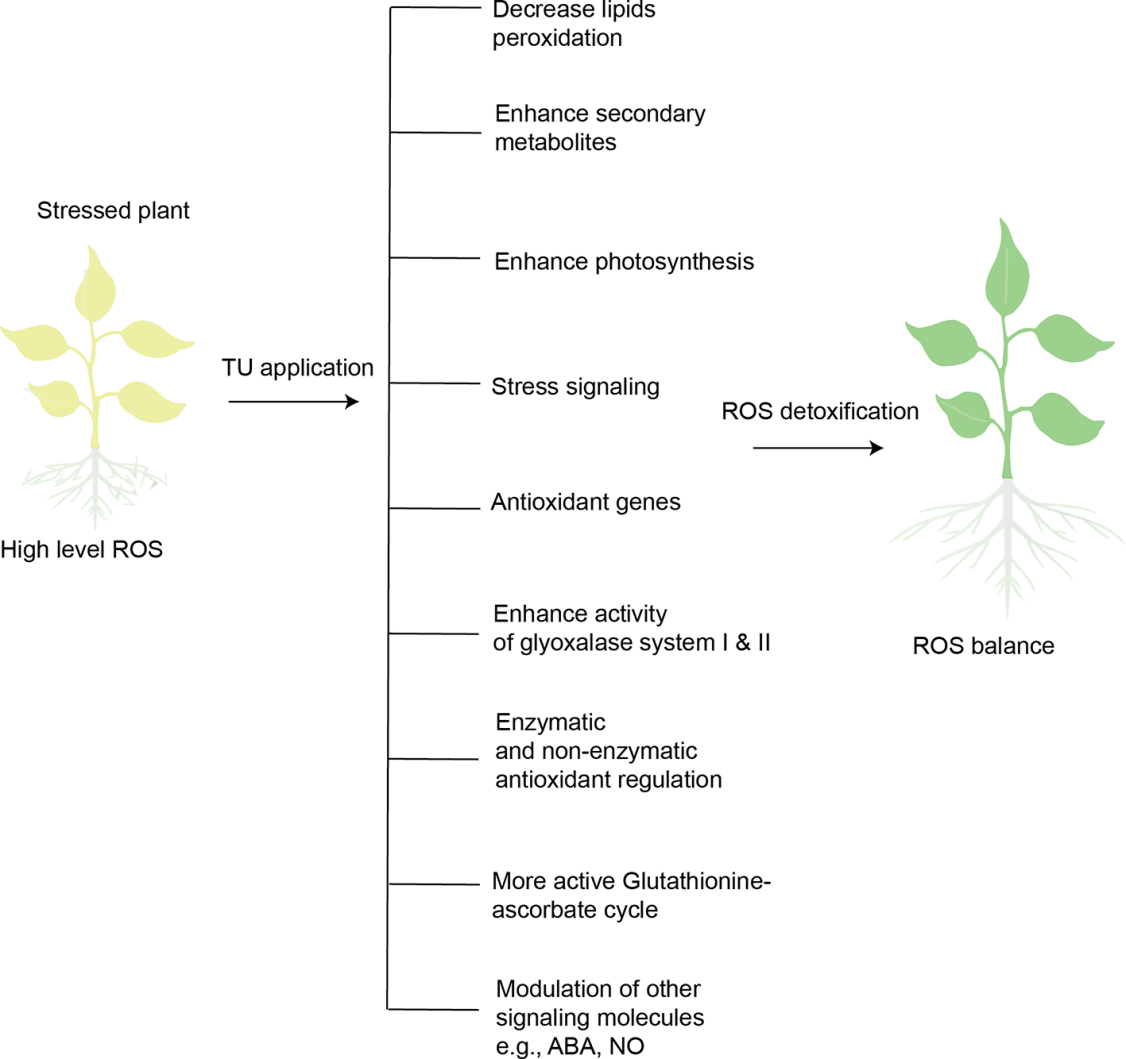
Overview of TU-mediated ROS-detoxification in stressed plants. Plants exposed to abiotic stresses (heat, cold, drought, salinity, heavy metal stress) increase ROS levels. High-level ROS can disturb normal cell functioning, ultimately leading to cell death. TU application is capable of detoxifying ROS in stressed plants through the mechanisms illustrated.

Salicylic acid (another plant growth regulator) has been extensively reported to contribute to antioxidant metabolism, ROS-signaling, and ROS detoxification (Hernández et al., 2017). TU alleviated the negative impact of heat stress on oil content and photosynthesis of sunflower by inducing antioxidant defense (SOD, CAT, and APX), and maximizing these effects by improving biosynthesis of salicylic acid (Akladious, 2014). TU also involves in defense-related transcription reprogramming in plants. TU-mediated expression of genes related to antioxidant defense (Cu/Zn SOD) and metabolite biosynthesis (CAH) was observed in *Capsicum* seedlings (originated from TU treated seeds) exposed to chilling stress (Patade et al., 2012). In sum, TU application detoxifies ROS to strengthen antioxidant defense in stressed plants by integrating and optimizing defense circuits, including stress signaling, genetic expression, photosynthesis, enzymatic and non-enzymatic antioxidants, metabolic processes, and biosynthesis of secondary metabolites (**Figure 3**). Plants must increase the concentration and activity of antioxidant enzymes to survive in a stressed environment, which imposes significant energy costs. The preceding evidence suggests that exogenous supply of TU enhances antioxidant defense while reducing the metabolic cost.

### TU Interactions With Secondary Metabolites

Most plants divert a reasonable proportion of photo-assimilates to the synthesis of defense molecules that may have no apparent role in plant metabolism. These molecules are known as secondary metabolites. There are three kinds of secondary metabolite, including phenolics, terpenes, and N-containing compounds such as alkaloids, cyanogenic glycosides, glucosinolates, non-protein amino acids. Initially, the adaptive significance of these molecules was unidentified, but they have now been recognized to play a role in plant tolerance against environmental stresses (Bartwal et al., 2013; Böttger et al., 2018).

Several reports indicate the involvement of TU in the introduction of secondary metabolite synthesis in stressed plants (Hassanein et al., 2015; Perveen et al., 2016; Waqas et al., 2017). TU-induced increase in glucosinolate contents in canola was observed under sulfur deficiency (ur Rehman et al., 2013). Carotenoids (Car) pigments are secondary metabolites of isoprenoid origin and are involved in many defense mechanisms, such as membrane stability, light-harvesting, and ROS balance (Uarrota et al., 2018). TU application significantly improved Car content in maize and enhanced photosynthetic and transpiration rates (Perveen et al., 2015). TU-induced increase in secondary metabolites is not only limited to stress conditions but also occurs under normal field conditions. TU supplementation (1,000 mg l^-1^) significantly enhanced secondary metabolites and improved faba bean seed yield and protein content (Amin et al., 2014). In another study, TU (250, 500 mM) also improved carotenoid, ascorbic acid, polyphenols and nitrate contents in potato crop (Mani et al., 2014). Moreover, enhanced ultraviolet-B tolerance in Indian mustard was induced by TU-induced synthesis of vital secondary metabolites (including anthocyanin, flavonoid, and phenolic compounds) leading to lesser damage to leaf chlorophyll contents under UV-B stress (Pandey et al., 2012). TU seed treatment (10 mM) improved the activity of the important enzyme phenylalanine ammonia lyase activity in heat-stressed sunflower (Akladious, 2014), an enzyme which has a vital role in the biosynthesis of numerous secondary metabolites (Mrázová et al., 2017). Under salt stress, TU-induced increase in phenolic contents contributed to enhanced tolerance in mung bean plants (Perveen et al., 2016). Phenolics provide substrates for flavonoid biosynthesis. TU also induces the expression of genes (such as *pal* and *chs*) involved in biosynthesis of secondary metabolites (Pandey et al., 2012). Future research needs to look in detail at molecular mechanisms underlying TU-induced biosynthesis and metabolism of secondary metabolites and their transport.

### TU Influences on Hormones

TU can modulate numerous aspects of growth and development of agronomic and horticultural crops under normal conditions as well as stressed environments through interactions with other phytohormones (Abdelkader et al., 2012; Patel et al., 2016; Srivastava et al., 2017). Interactions between TU and other phytohormones, such as salicylic acid (Abdelkader et al., 2012), benzoic acid and polyamines (Hassanein et al., 2015), abscisic acid, auxin and jasmonates (Srivastava et al., 2017) are already well known. There are two modes of TU interaction with phytohormones and other PGRs. Either exogenous application of TU enhances the endogenous levels of phytohormones, such as abscisic acid, or TU interacts positively when applied in combination with other PGRs, and phytohormones lead to improved performance of stressed plants. However, optimized dosage is essential. For example, a TU-mediated rise in the endogenous level of salicylic acid contributed to reversing the impacts of mild and high temperature stress (35°C and 45°C) in sunflower (Akladious, 2014). Moreover, the combined application of SA and TU interacted positively in terms of improved yield and nutritional contents of grains in drought-stressed wheat by tightly controlling oxidative damage (Hassanein et al., 2015). Benzoic acids are vital structural elements for numerous essential plant hormones (Widhalm and Dudareva, 2015). Externally applied benzoic acid and TU increased wheat crop productivity and grain nutrition by improving photosynthetic pigments and maintaining flag leaf greenery at critical growth stages (Amin et al., 2016). Gooseberry (*Emblica officinalis* Gaertn) shrubs often face heavy fruit drop, which significantly reduces yield. TU treatment in combination with gibberellic acid dramatically enhanced fruit quality parameters (ascorbic acid, sugars contents, and acidity) and fruit setting (Anshuman and Singh, 2015). TU application also influenced the biosynthesis of numerous hormones including an increase in the level of cytokinins (zeatin, zeatin riboside), gibberellins, Indole-3-Acetic acid, and decreased the growth inhibitory hormone abscisic acid under both normal and stressed conditions (Abdelkader et al., 2012).

TU exhibits an ubiquitous role in inducing biosynthesis of phytohormones. TU-induced expression of *PHO1* (phosphate exporter) and *BRX4* (from Brevis-radix family, considered as a vital gene family for maintaining plant abilities under stress conditions) integrates signaling cross-talks and triggers biosynthesis of numerous phytohormones including auxin, brassinosteroid, and abscisic acid (Beuchat et al., 2010). Moreover, TU regulates post-transcriptional changes to increase the expression of these genes (Srivastava et al., 2017). Concurrently, levels of hormones are synchronized to maximize TU-mediated ameliorative effects in stressed plants. For instance, in response to TU application in salt-stressed plants, ABA level was modulated for a short period, which induced the jasmonates (jasmonic acid, and methyl jasmonate) to select defense overgrowth under stress. A higher level of auxin also contributed to defense. This synchronization was reflected in overall improved growth (Srivastava et al., 2017). Thus, hormone-based regulation and signaling cross-talk in response to externally applied TU is a critical mechanism to induce tolerance against environmental stresses.

### Conclusion and Future Needs

One of the biggest challenges in agricultural production is to guarantee current and future food security. However, environmental stresses are a significant hurdle in this endeavor. Several physiological, morphological, and molecular mechanisms are involved in stress tolerance of crop plants. Research conducted over recent decades has clearly demonstrated that TU can be used as a practical solution to mitigate the adverse effects of abiotic stresses and sustain productivity. Interaction of different intricate tolerance mechanisms is crucial for crop plants to deal with stress factors. TU enhances nutrient uptake, and modulates the biosynthesis of numerous secondary key metabolites, osmolytes, and plant hormones, and regulates various metabolic activities to induce tolerance under environmental stresses. TU-mediated benefits are not restricted to stress conditions, but also improve plant performance in normal conditions, using the same principal mechanisms.

However, there is still much that needs to be further explored. For instance, TU application affects the expression levels of a number of defense genes (*CaWRKY30*, *Cu/Zn SOD*, *PROX1*, *Osmotin, CAH*) in cold-stressed plants. However, more detailed molecular work is needed on cross-talking TU-responsive genes and their potential in inducing tolerance against other abiotic stresses. Likewise, more detailed research needs to be done on the role of TU in plant signaling networks under stress. Exhaustive genomic, proteomic, and histology studies can reveal the TU-responsive genetic networks and proteins under stresses. TU's role as synchronizer between different plant processes, such as hormonal balance, signaling cross-talk, growth, production of secondary metabolites, and assimilate portioning needs to be further validated in a wide range of environmental conditions and crops. The studies reviewed in this paper show that TU increases the nutritional quality of several crop types including lentil, maize, wheat, and onion. TU may, therefore, be critical in the future to cope with the challenges to human nutrition amidst ongoing global climate change (Smith and Myers, 2018). Therefore, TU can also be tested under FACE (free-air carbon dioxide enrichment) experiments to evaluate its role in influencing nutritional quality under anticipated elevated temperature and CO_2_ concentration. Moreover, TU increases nutrient uptake in plants. Therefore, the effects of TU combination with a variety of soil amendments, such as biochar or bagasse compost, can be evaluated in different pedoclimatic conditions. Considering ongoing climate change, future studies may be conducted to develop further technologies, including TU mixtures with other PGRs to induce more tolerance against abiotic stresses.

## Author Contributions

MAW conceptualized the idea and wrote the first draft. CK, AR, IN, AW, YL helped in writing, reviewing, and further improvement in the manuscript. MF provided the technical guidance.

## Conflict of Interest

The authors declare that the research was conducted in the absence of any commercial or financial relationships that could be construed as a potential conflict of interest.
